# Microvascular heterogeneity exploration in core and invasive zones of orthotopic rat glioblastoma via ultrasound localization microscopy

**DOI:** 10.1186/s41747-025-00555-4

**Published:** 2025-03-05

**Authors:** Xing Hu, Gaobo Zhang, Yong Wang, Xiandi Zhang, Rong Xie, Xin Liu, Hong Ding

**Affiliations:** 1 Department of Ultrasound, Huashan Hospital, Fudan Univertity, Shanghai, China; 2 Department of Biomedical Engineering, School of Information Science and Technology, Fudan University, Shanghai, China; 3 Department of Neurosurgery, Huashan Hospital, E Fudan Univertity, Shanghai, China; 4 Academy for Engineering and Technology, Fudan University, Shanghai, China

**Keywords:** Glioblastoma, Heterogeneity, Invasive, Microvascular, Ultrasound localization microscopy

## Abstract

**Background:**

We studied the microvascular structure and function of *in situ* glioblastoma using ultrasound localization microscopy (ULM).

**Methods:**

The *in vivo* study was conducted via craniotomy in six Sprague–Dawley rats. Capillary pattern, capillary hemodynamics, and functional quantitative parameters were compared among tumor core, invasive zone, and normal brain tissue with *ex vivo* micro-computed tomography (micro-CT) and scanning electron microscopy. Correlations between quantitative parameters and histopathological vascular density (VD-H), proliferation index, and histopathological vascular maturity index (VMI-H) were evaluated. Kruskal–Wallis *H*, ANOVA, Mann–Whitney *U*, Pearson, and Spearman correlation statistics were used.

**Results:**

Compared to the tumor core, the invasive zone exhibited higher microvascularity structural disorder and complexity, increased hemodynamic heterogeneity, higher local blood flow perfusion (*p* ≤ 0.033), and slightly lower average flow velocity (*p* = 0.873). Significant differences were observed between the invasive zone and normal brain tissue across all parameters (*p* ≤ 0.001). ULM demonstrated higher microstructural resolution compared to micro-CT and a nonsignificant difference compared to scanning electron microscopy. The invasive zone vascular density correlated with VD-H (*r* = 0.781, *p* < 0.001). Vessel diameter (*r* = 0.960, *p* < 0.001), curvature (*r* = 0.438, *p* = 0.047), blood flow velocity (*r* = 0.487, *p* = 0.025), and blood flow volume (*r* = 0.858, *p* < 0.001) correlated with proliferation index. Vascular density (*r* = -0.444, *p* = 0.044) and fractal dimension (*r* = -0.933, *p* < 0.001) correlated with VMI-H.

**Conclusion:**

ULM provided high-resolution, noninvasive imaging of glioblastoma microvascularity, offering insights into structural/functional abnormalities.

**Relevance statement:**

ULM technology based on ultrafast ultrasound can accurately quantify the microvessels of glioblastoma, providing a new method for evaluating the effectiveness of antiangiogenic therapy and visualizing disease progression. This method may facilitate early therapeutic assessment.

**Key Points:**

- ULM reliably captures the vascular structures and hemodynamic features of glioblastoma in rats.
- Micro-CT and scanning electron microscopy validated its effectiveness in microvascular non-invasion characterization.
- ULM is expected to effectively evaluate glioblastoma anti-vascular therapy response.

**Graphical Abstract:**

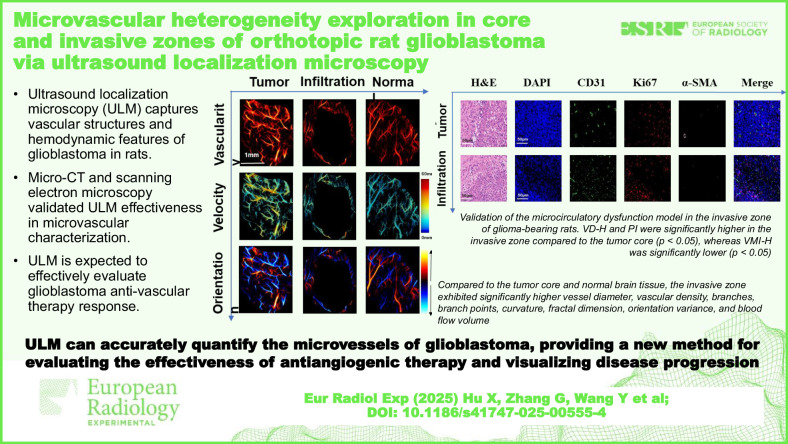

## Background

Glioblastoma multiforme (GBM) is the most prevalent malignant brain tumor, constituting 50% of primary malignant central nervous system tumors and 57% of gliomas, with a median survival of 12.2–18.2 months [1, 2]. Tumor-induced pathological neovascularization in adjacent tissues involves vascular dilation, tortuosity, abnormal branching, and arteriovenous shunting. GBM is characterized by dysplastic vessel walls, discontinuous endothelial cell linings, and increased microvascular density at the margins, facilitating cell diffusion and invasion [3, 4]. Disruption of capillary pattern (CP) and capillary hemodynamics (CH) is crucial for disease progression, serving as fundamental structures and invasion substrates for tumors and indicating sensitivity to antiangiogenic therapy [5, 6]. Tumor microenvironment heterogeneity, particularly microvascular (MV) heterogeneity, significantly contributes to GBM treatment failure [7]. Comprehensive characterization of MV systems can elucidate their role in GBM pathogenesis, underscoring the necessity of detailed exploration of invasive zone CP and CH heterogeneity essential for clinical management [8]. Constructing invasive molecular models is challenging due to the human brain’s complex structure and cellular diversity, particularly its three-dimensional architecture [9]. Detailed microanatomical studies, including those of vascular niches, are vital for understanding tumor biology and potentially offering treatment insights.

The GBM invasive region, radiologically defined as the area outside the magnetic resonance imaging (MRI) T1-weighted contrast-enhanced image, is the most active and functionally significant part of the tumor. Studies suggest that the area displayed by the fluid-attenuated inversion recovery sequence represents metastasis or recurrence sites, which are possibly not contrast-enhanced but pathologically infiltrative, and often more active and invasive than contrast-enhanced tumor regions [10, 11]. Combining multiple imaging sequences significantly enhances the ability to identify the invasive zone [12]. Fluoroethyl-l-tyrosine positron emission tomography tracers, unrelated to blood-brain barrier disruption, show promise in identifying non-contrast-enhanced invasive tissues, but are limited by spatial resolution [13]. Techniques such as multiphoton fluorescence microscopy, optical coherence tomography, and photoacoustic microscopy reveal capillary-level morphological and fractal characteristics but are limited to surface imaging and histopathological evidence [14]. Cryo-imaging visualizes single fluorescently labeled cells through thousands of stitched images but lacks dynamic and longitudinal monitoring capabilities [15]. GBM progression results in neurovascular uncoupling and blood-brain barrier dysfunction, affecting data accuracy [16]. Brain slice histopathology reveals MV morphology and invasion but lacks critical hemodynamic damage information. Even high-field MRIs’ submillimeter spatial resolution is insufficient for MV imaging, necessitating high-resolution *in vivo* imaging to map MV structural and functional disruptions, thus better elucidating GBM invasive behavior.

Ultrafast Doppler, utilizing ultrafast ultrasound technology and plane wave imaging, scans up to 20,000 frames/s, significantly enhancing blood flow detection sensitivity and offering a high spatiotemporal resolution of CP and CH imaging [17]. Ultrasound localization microscopy (ULM) employs high dilution and extended duration separation of contrast agent echoes, accurately quantifying local velocity [18]. The singular value decomposition filtering method extracts feature vectors of tissue, microbubble, and noise signals, optimizing parameters to mitigate signal-to-noise ratio and resolution losses caused by non-focused ultrasound beams. This emerging technology balances spatial resolution and penetration depth, thereby enhancing imaging quality and vascular presentation [19].

In this study, we initially compared vascular structures and hemodynamic changes in the tumor core, invasive zone, and normal brain tissue to reliably capture MV characteristics and provide a comprehensive perspective for heterogeneity assessment. Subsequently, we compared ULM with traditional imaging methods, including micro-computed tomography (micro-CT) and scanning electron microscopy (SEM), to advance its clinical application for noninvasive glioma angiogenesis assessment. Finally, we analyzed the correlation between ULM-derived data from the invasive zone and histopathology indices, including histopathological vascular density (VD-H), proliferation index (PI), and histopathological vascular maturity index (VMI-H), further validating ULM’s value in MV characterization. We anticipated this approach may become a valuable tool for studying GBM antiangiogenic therapy responses [20].

## Methods

### Ethics approval

**Fig. 1:**
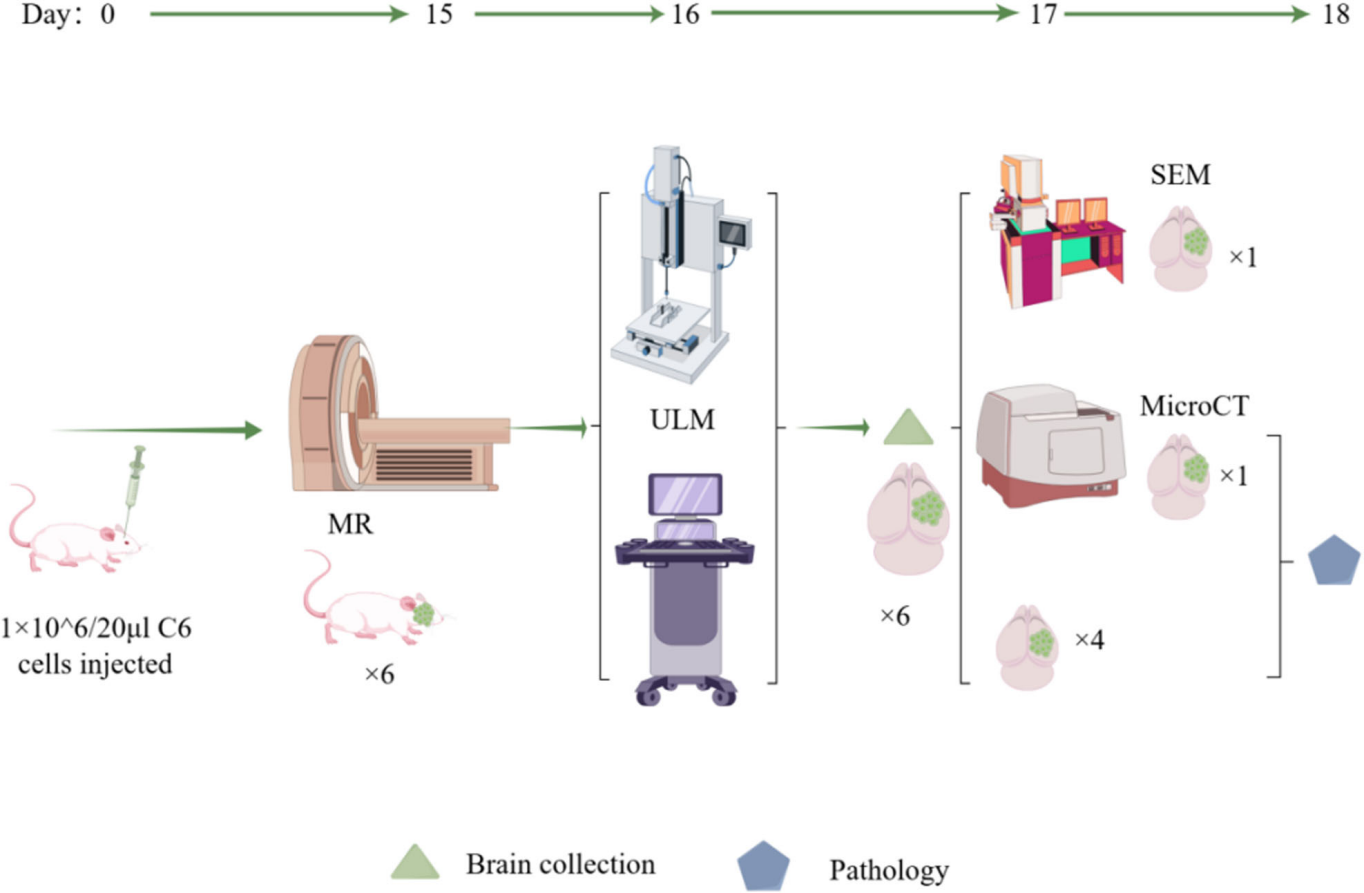
Research workflow diagram

All experiments complied with the ARRIVE guidelines and were approved by the Animal Ethics Committee of Fudan University (202408008S). The research workflow is shown in Fig. 1.

### Animal model

Six male Sprague–Dawley rats (Jiangsu KeyGEN BioTECH, China; Gpt, outbred, D000017), 6-weeks-old, weighing 200–250 g, were used to induce the GBM model. The rats were housed in a temperature- and humidity-controlled room with a 12-h light/dark cycle and given free access to standard rodent food and sterilized tap water. The C6 rat glioma cell line was obtained from the National Institutes of Health (USA) and cultured in DMEM with 10% fetal bovine serum and 5% penicillin–streptomycin.

C6 cells were prepared at a concentration of 5 × 10^5^/10 μL. The rats were anesthetized with an intraperitoneal injection of 60 mg/kg of ketamine and xylazine. The rats’ heads were fixed in a stereotactic frame. A 25-gauge flat-tip syringe (Hamilton Company, Reno, Nevada, USA) was used to inject 20 μL of the cell suspension into the caudate nucleus. The procedure involved making a 0.5-cm longitudinal incision on the scalp, exposing the bregma and sagittal suture. The injection site was 3 mm lateral to the sagittal suture and 1 mm anterior to the coronal suture, with a needle depth of approximately 5.5 mm and an injection rate of 4 μL/min. The needle remained for 2 min before slowly withdrawing. The injection site was sealed with bone wax, and the scalp was sutured.

### MRI protocol

On day 15 after cell implantation, MRI scans were conducted on rats under mild anesthesia with isoflurane (1–1.25% oxygen concentration) using a 3.0-T MRI (uMR870, United Imaging Healthcare, Shanghai, China) in the coronal plane. The protocol included unenhanced T2-weighted, fluid-attenuated inversion recovery, diffusion-weighted sequences, and T1-weighted contrast-enhanced imaging following the tail vein injection of 0.6 mmol/kg gadopentetate dimeglumine (Bayer HealthCare, Leverkusen, Germany) (Supplementary Table S1).

### ULM protocol

#### Surgical area preparation

On day 16, rats were anesthetized in a gas induction chamber (R500, RWD Life Science, Shanghai, China) with 4% isoflurane (R510-22-10, RWD) and medical-grade oxygen and maintained at 1% concentration. The neck hair was shaved, and the skin was disinfected with iodine and 70% ethanol. Rats were placed in the supine position for right jugular vein catheterization using a medical-grade polyethylene catheter PE10 (inside diameter 0.28 mm, outside diameter 0.61 mm). An ophthalmic solution was applied to prevent eye dryness., and the head was fixed to a stereotactic frame. Following a scalp incision, the periosteum was removed with a scalpel, and the skull was thinned with a cranial drill (78001, RWD) until the vascular system was visible. Under a microscope (DOM-1001, RWD), the skull was carefully opened using microsurgical forceps while ensuring the integrity of the larger blood vessels and the superior sagittal sinus. Minor bleeding from superficial injuries was controlled with hemostatic sponges, and sterile saline was used to prevent thermal injury, reduce bleeding, and minimize swelling.

#### Multiplane radiofrequency data acquisition

The L22-14vX LF transducer (15.625 MHz, MS200, VisualSonics Ltd., Toronto, ON, Canada) was mounted on a three-dimensional-printed, high-precision translation motor (VT-80 linear stage, Physik Instrumente, Auburn, MA, USA), which was fixed to the stereotactic imaging frame for coronal brain slices. Tumor position was initially marked using the Mindray M10 Portable Ultrasound System (Shenzhen Mindray Bio-Medical Electronics Co., Ltd., China) and coregistered with MRI coronal slices. The Verasonics Vantage 256 system (Verasonics Ltd., Kirkland, WA, USA) and the L22-14vX-LF transducer facilitated 1 mm linearly incremented movements. Diluted SonoVue (Bracco Imaging, Massy, France) was administered at 80 μL/min using a programmable microsyringe pump (R462, RWD). The imaging sequence comprised multiple 5-angle plane waves (-5°, -2.5°, 0°, 2.5°, and 5°) [21]. Data acquisition was synchronized using a function generator and processed in MATLAB (The MathWorks, Natick, MA, USA, R2020a). The number of scanned slices per rat, according to tumor size, is shown in Supplementary Table S2.

Singular value decomposition filtering was utilized to extract microbubble signals from the tissue background of each in-phase/quadrature data set. The optimal clutter filter threshold was determined at the inflection point of the singular value curve. Microbubble centroids were identified using MATLAB’s radial symmetry algorithm, and the Hungarian algorithm was applied for frame-to-frame matching and trajectory. A minimum microbubble trajectory length of 15 frames was enforced, and each acquisition was aggregated to produce a ULM reconstruction of the vascular system. Due to significant echo scattering from ear bars or acoustic shadowing from the remaining lateral skull parts, the reconstruction of the outermost cortical vessels was suboptimal [22].

### Micro-CT protocol

A rat underwent a midline abdominal incision to expose the heart. A venous infusion needle was inserted into the left ventricle, approximately 2–3 mm deep, and secured with hemostatic forceps. The right atrium was incised, and the heart was perfused with 120 mL of 37 °C saline containing 10 IU/mL low molecular weight heparin, followed by 120 mL of 4% paraformaldehyde, and finally thoroughly rinsed with saline. Approximately 100 mL of MICROFIL^®^ MV-120 Blue (Flow Tech, Carver, MA, USA) was perfused at a rate of 5 mL/min, after which the left ventricle and right atrium were ligated. The rat was positioned head-down at 4 °C overnight. The brain was then removed intact, fixed with 10% neutral formalin, and scanned in the SkyScan1276 micro-CT (Bruker Corporation, Billerica, MA, USA) at 29 kV, 169 µA, and a resolution of 8.8 µm [23]. A three-dimensional reconstruction was generated slice by slice, with a 1-mm thickness for comparison with ULM.

### SEM

After data collection, the rat was euthanized, and a tumor sample (10 × 8 × 1 mm^3^) was taken, cleaned with saline, prefixed in 1.25% glutaraldehyde at 4 °C for 2 h, washed three times in 0.1 M phosphate buffer (15 min each), and fixed in 1% osmium acid at 4 °C for 2 h. The sample was dehydrated stepwise in increasing concentrations of ethanol (50%, 60%, 70%, 80%, and 90%) and then transitioned to ethanol and amyl acetate. The sample was placed in a critical point dryer (Hitachi Critical Point Dryer-2, Hitachi High-Tech Corporation, Tokyo, Japan) for 2 h. The dried sample was adhered to a sample stage with double-sided tape and sputter-coated with gold for 3 min in an Ion Beam-3 ion sputterer. Vascular morphology was observed using a Hitachi SU-8010 (Hitachi High-Tech Corporation, Tokyo, Japan) and compared with ULM [24].

### Histopathologic analysis

The tumor location was coregistered using the Mindray M10 Portable Ultrasound System (Shenzhen Mindray Bio-Medical Electronics Co., Ltd., China) and sliced into 1-mm thick slices using a Rat Brain Matrix (World Precision Instruments-RBMA-600S, Sarasota, Florida, USA). The tissue was decalcified in 10% ethylenediaminetetraacetic acid and embedded in paraffin. Sections were cut into a 5-μm thickness and stained with hematoxylin and eosin for histopathological examination. Whole slide images were captured at 40× magnification (0.25 μm/pixel) using the (3D HISTECH Ltd., Budapest, Hungary) and stored in “Slide and Viewable Storage” format. Visualization was performed at 200× magnification using the MAGSCAN-NER scanner (KF-PRO005, Konfoong Bioinformation Technology Co., Ltd, Seoul, South Korea), and the digital scans were analyzed using Tissue Studio^®^ software (Definens, Munich, Germany). Based on histopathological characteristics, the tissue was categorized into three types: tumor core (≥ 90% tumor cells), invasive (< 90% tumor cells), and normal (no tumor cells).

Rat anti-mouse CD31 (Abcam, AB222783, Abcam plc, Cambridge, United Kingdom) was used for endothelial cell immunostaining to analyze vascular density. Rabbit polyclonal anti-Ki67 (Abcam, AB16667) stained proliferative cells. Biotinylated anti-α-smooth muscle actin (Abcam, AB124964) labeled smooth muscle cells and pericytes for vascular maturity analysis. The cell nuclei were stained with 4,6-diamidino-2-phenylindole. VD-H was calculated by dividing the area of CD31-positive structures by the manually selected tumor region area. The ratio of CD31- and αSMA-positive areas to CD31-only positive areas determined the VMI-H.

### Imaging parameter evaluation

For each slice, all other sequences (T1-weighted, T2-weighted, diffusion-weighted imaging, and T1-weighted contrast-enhanced) were co-registered with the fluid-attenuated inversion recovery sequence. Magnetic field inhomogeneities were corrected using the bias field correction tool in the FMRIB Software Library−FSL, and rigid registration was performed using the FMRIB’s Linear Image Registration Tool−FLIRT. In the contrast-enhanced sequence, the contrast-enhanced tumor region, including necrosis, was considered the region of interest, while in the fluid-attenuated inversion-recovery sequence, regions with high or abnormal signals (including tumor and edema) were selected. The region of interest was manually delineated based on registration results and mapped to the super-resolution image, covering the tumor core, invasive zone, and normal tissue. Image features were extracted using the scale-invariant feature transform and wavelet transform algorithms in Fiji/ImageJ v.1.54f software (Wayne Rasband, National Institutes of Health, USA) and segmented using the Weka Trainable Segmentation.

Vessel segmentation was performed in a supervised, semiautomated manner. A random forest classifier model was trained, incorporating image preprocessing, deep learning-based vessel segmentation, feature extraction, and scan modulation. Vessel area, skeleton, and midpoints were extracted from binary images as a two-dimensional representation of the three-dimensional volume. The out-of-bag error, indicating the error rate of samples not used to train decision trees, was used for internal unbiased estimation. To enhance the model’s generalization ability, the out-of-bag error was maintained below 5%.

Curvature is defined as:

1 $${{\rm{Curvature}}}=\frac{L{{\rm{c}}}}{L}$$

*Lc*: actual path length of a vessel segment; *L*: linear distance between the segment endpoints.

The fractal dimension measures the complexity and branching pattern of the vascular network, indicating their branching and twisting. Higher values indicate more complex vascular networks. The box-counting method was employed for segmentation at different scales, and linear regression determined the fractal dimension. To capture image fractal features, a suitable two-dimensional Euclidean space with *R*² > 0.99 was selected to ensure a good fit between the model and actual data:

2 $${{\rm{Fractal\; dimension}}}={{\mathrm{lim}}}_{r\to \infty }\frac{\log M\left(r\right)}{\log \left({{\rm{l}}}/r\right)}$$

*r* is the scale of the grid, and *M*(*r*) is the minimum number of grids covering the fractal.

Orientation variance can be measured by the direction of the vessels. Assuming the average vector of vessel direction is $\bar{{\theta }}$, orientation variance can be calculated as follows:

3 $${{\rm{Orientation}}}\,{{\rm{variance}}}=\frac{1}{N}{{\sum }_{i=1}^{N}({\theta }_{i}-\overline{\theta })}^{2}$$

where *N* is the number of vessels, *θi* is the direction of the *i*-th data point, and $\overline{\theta }$ is the average direction of all angles.

Velocity and volume reflect the perfusion status and are calculated under the assumption of Poiseuille flow:

4 $${{\rm{Q}}}={{\rm{A}}}\times {{\rm{V}}}$$

where *Q* is the blood flow volume, *A* is the cross-sectional area of the vessel, and *V* is the blood flow velocity.

### Statistical analysis

Statistical analyses were performed using Origin 2021 (OriginLab Corporation, Northampton, MA, USA) and GraphPad Prism 10 (GraphPad Software, Inc., San Diego, CA, USA). All repeated measurements from the same animal (*e.g*., multiple histopathological sections, imaging planes, and vascular profiles) were averaged into a single variable, with data presented as mean ± 95% standard error of the mean. Kolmogorov–Smirnov was used to assess the normality of data distribution. The Kruskal–Wallis *H-*test, or ANOVA with Bonferroni correction, was used for multiple comparisons. The Mann–Whitney *U*-test evaluated differences in vascular markers between tumor and invasive zones, and box plots were used to illustrate differences. Pearson correlation analysis was conducted for normally distributed data, while Spearman rank correlation was used for non-normally distributed data. A correlation value (*r*) of 0.400–0.699 was considered moderate, and greater than 0.700 was considered strong. Significance levels were set as ^ns^*p* ≥ 0.05, *p < 0.05, **p < 0.01, and ***p < 0.001.

## Results

### ULM differentiation of tumor microcirculation morphology and function

**Fig. 2:**
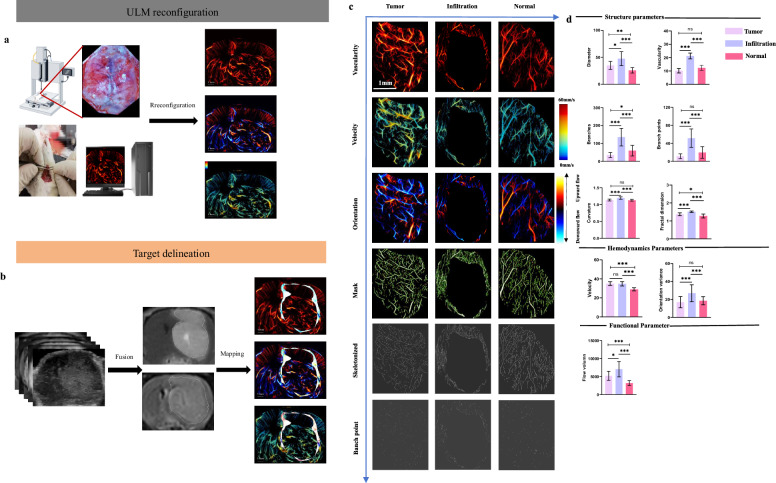
**a** After craniotomy, rats were secured in a stereotactic frame using ear bars. Microbubbles were injected through the jugular vein, and an ultrasound transducer with a high-precision motor was positioned to acquire anatomical imaging planes of interest. **b** The tumor core was delineated based on T1-weighted contrast-enhanced enhancement. After coregistration of multi-sequence MRI and ultrasound images, the invasive zone was manually delineated and mapped onto super-resolution morphology, velocity, and directional maps. The shaded white area represented the invasive zone. **c** The results after image segmentation include effective visualization of vascular skeletonization, branches, and branch points. **d** The parameter analysis includes structural, hemodynamic, and functional parameters for the tumor core, invasive zone, and normal brain tissue

Our analysis revealed that, compared to the tumor core (*p1*) and normal brain tissue (*p2*), the invasive zone exhibited significantly higher vessel diameter (*p1* = 0.012, *p2* < 0.001), vascular density (*p1* < 0.001, *p2* < 0.001), branches (*p1* < 0.001, *p2* < 0.001), branch points (*p1* < 0.001, *p2* < 0.001), curvature (*p1* < 0.001, *p2* < 0.001), fractal dimension (*p1* < 0.001, *p2* < 0.001), orientation variance (*p1* < 0.001, *p2* < 0.001), and blood flow volume (*p1* = 0.033, *p2* < 0.001), whereas the average velocity was lower but not statistically significant (*p1* = 0.873, *p2* < 0.001) (Fig. 2 and Supplementary Table S3).

### Comparison of micro-CT and ULM

**Fig. 3:**
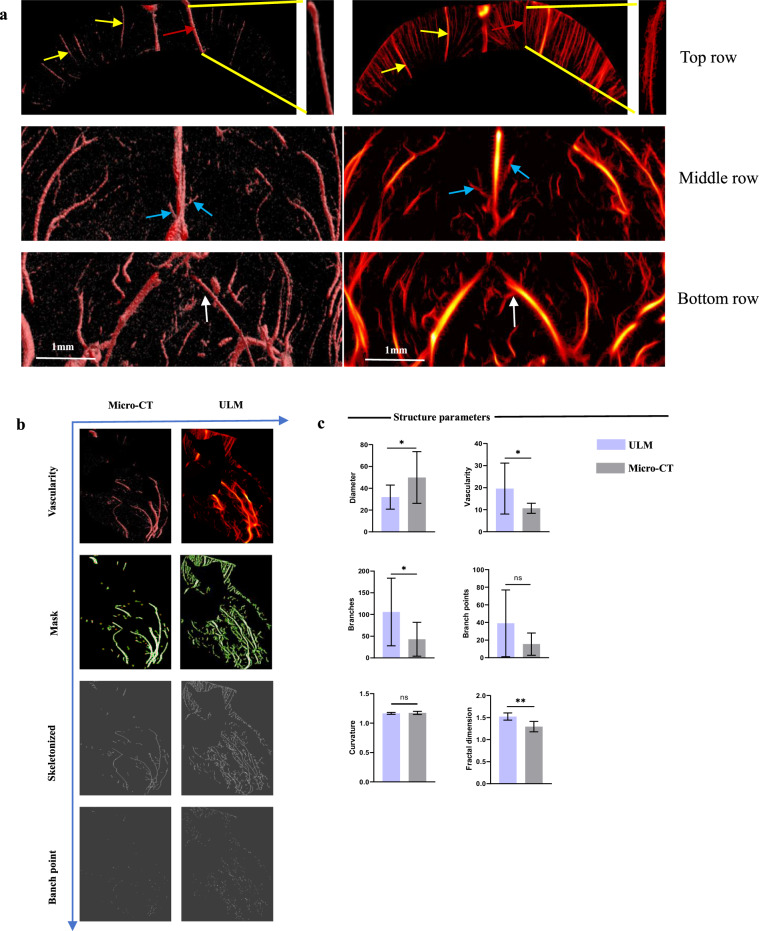
**a** Top row: comparison of micro-CT and ULM microstructures. No difference was observed in large blood vessels (yellow arrow); however, ULM exhibited higher resolution for microstructures, particularly for identifying adjacent vessels (red arrow). Middle row: ULM displayed distal vessel structures, with some branches visible (blue arrow), demonstrating continuity between distal and proximal vessels. Bottom row: ULM provided a more detailed visualization of branching structures, including sensitivity to tiny distal branches (white arrow). **b** The segmentation results of micro-CT and ULM provided visualization of the vascular skeleton and branches in the invasive zone. **c** Quantitative comparison of structural parameters between micro-CT and ULM. Micro-CT, Micro-computed tomography; ULM, Ultrasound localization microscopy

In the invasive zone, compared to micro-CT, vessel diameter significantly decreased (*p* = 0.041), whereas vascular density (*p* = 0.031), branches (*p* = 0.036), and fractal dimension (*p* = 0.002) significantly increased. No significant differences were observed in branch points (*p* = 0.064) and curvature (*p* = 0.643) (Fig. 3 and Supplementary Table S4). Similar trends were observed in the tumor core (Supplementary Fig. S1 and Supplementary Table S5).

### Comparison of SEM and ULM

**Fig. 4:**
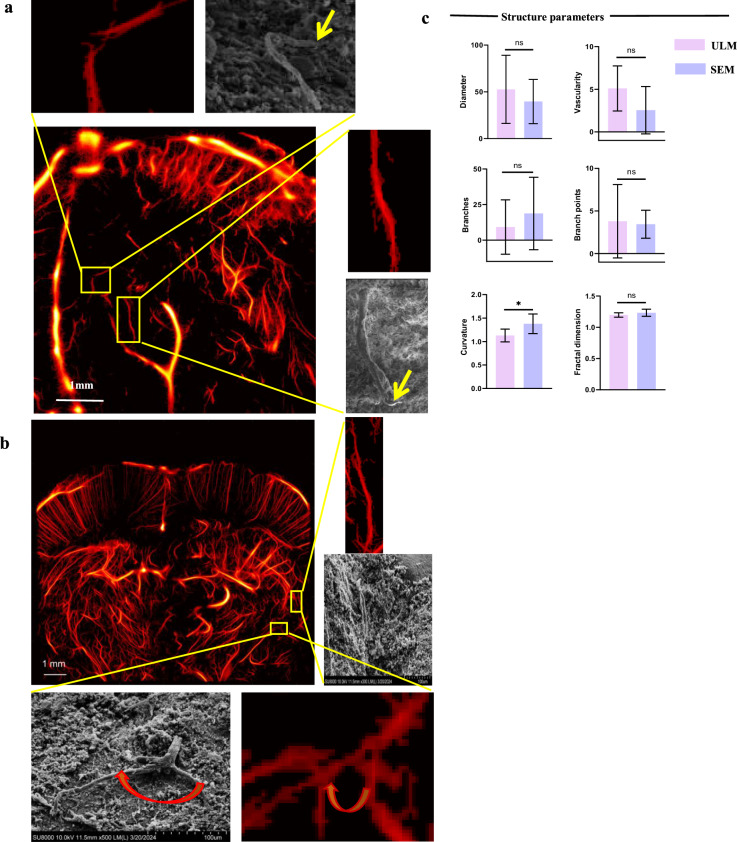
**a** Comparison of ULM and SEM. Both techniques showed similar vascular pathways. SEM, being a surface imaging technique, was unable to visualize deep vessels (yellow arrow). The white scale bar represents 1 mm. **b** During tissue preparation, dehydration, and fixation procedures caused vessel displacement and increased curvature, but the overall contour remained intact (red arrow). **c** The structural parameters (vascular density, curvature, branches, and branch points) showed no significant differences between SEM and ULM. SEM, Scanning electron microscopy; ULM, Ultrasound localization microscopy

Compared to SEM, ULM exhibited no significant differences in vessel diameter (*p* = 0.379), vascular density (*p* = 0.079), branches (*p* = 0.377), branch points (*p* = 0.813), and fractal dimension (*p* = 0.131). However, vessel curvature was significantly lower (*p* = 0.019) (Fig. 4 and Supplementary Table S6).

### Validation of microcirculatory dysfunction in the invasive zone

**Fig. 5:**
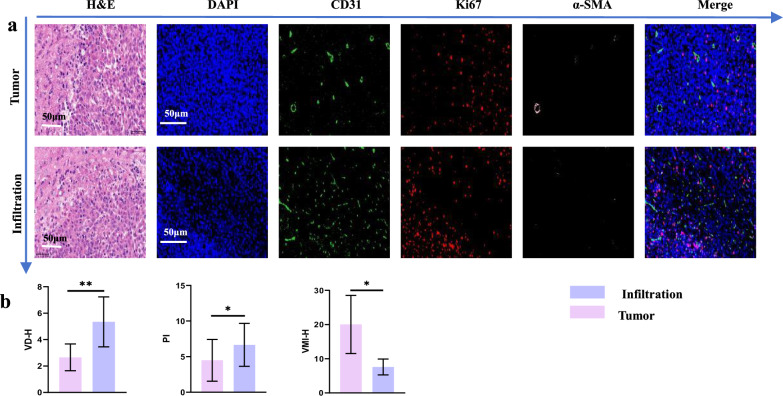
Validation of the microcirculatory dysfunction model in the invasive zone of glioma-bearing rats. **a** H&E staining, nuclear staining, CD31 staining, Ki67 staining, and α-SMA staining were performed. The white scale bar represents 50 μm. **b** Comparison of VD-H, PI, and VMI-H between the invasive zone and the tumor core. DAPI, 4’,6-diamidino-2-phenylindole; H&E, Hematoxylin and eosin; PI, Proliferation index; SMA, Smooth muscle actin; VD-H, Histopathological vascular density; VMI-H, Histopathological vascular maturity index

VD-H (*p* = 0.006) and PI (*p* = 0.030) were significantly higher in the invasive zone compared to the tumor core, whereas VMI-H (*p* = 0.019) was significantly lower (Fig. 5 and Supplementary Table S7).

### Correlation of histopathological assessment with ULM-derived heterogeneity indicators

**Fig. 6:**
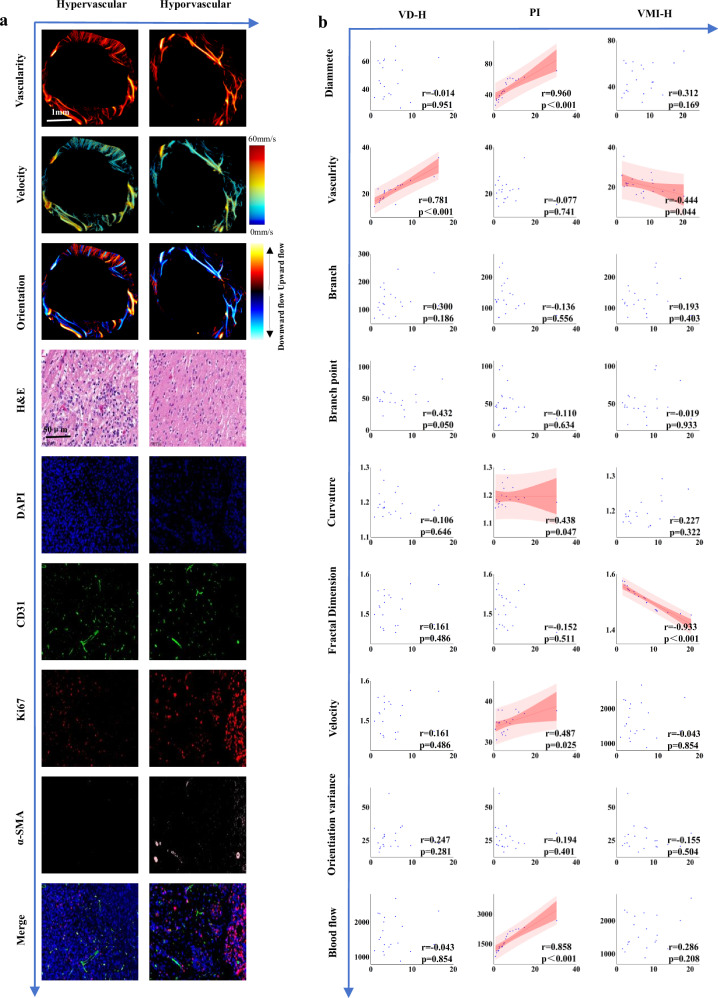
**a** Two representative tissue sections were shown. Left: high enhancement (high tumor density, high endothelial proliferation, low VMI-H). Right: low enhancement (low tumor density, low endothelial proliferation, high VMI-H). **b** Correlation between histopathological and ULM data, demonstrating significant correlations with linear fit and 95% confidence intervals. ULM, Ultrasound localization microscopy; VMI-H, Histopathological vascular maturity index

Vascular density exhibited a strong positive correlation with VD-H (*r* = 0.781, *p* < 0.001) and a moderate positive correlation with branch points (*r* = 0.432, *p* = 0.044). Vessel diameter (*r* = 0.960, *p* < 0.001) and blood flow volume (*r* = 0.858, *p* < 0.001) were strongly correlated with Ki67. Blood flow velocity (*r* = 0.487, *p* = 0.025) and curvature (*r* = 0.438, *p* = 0.047) demonstrated moderate positive correlations with PI, respectively. Fractal dimension exhibited a strong negative correlation with VMI-H (*r* = -0.933, *p* < 0.001) (Fig. 6 and Supplementary Table S8).

## Discussion

Incomplete surgical resection of the invasive zone often leads to high recurrence and mortality rates in GBM, with up to 77% of cases recurring within 2 cm of the original site. However, the invasive zone exists within a complex neural network, and expanding resection increases the risk of postoperative functional impairment, including aphasia and paralysis [25]. This is largely due to obstacles in intraoperative visualization of the invasive structure and functional areas during surgery. Brain microstructure complexity and tumor heterogeneity further complicate the functional localization of the invasive zone, presenting a significant clinical challenge [3]. Additionally, the tumor mass effect distorts anatomical relationships and disrupts cell interactions surrounding neovascularization, leading to neural network reconstruction and regional neurovascular coupling disruption. These factors undermine the reliability of preoperative functional assessments, reducing the accuracy of invasive zone localization and data [16]. Studies have shown that anatomical characterization of the invasive zone’s CP, CH, and microvascular perfusion can accurately depict functional area information [9]. In this study, leveraging the ultra-sensitive, high- resolution imaging capabilities of ULM, we effectively characterized the microstructural and hemodynamic features of GBM invasion.

To our knowledge, this is the first report on GBM’s vascular heterogeneity, emphasizing the significance of MV in the invasive zone. Dynamic contrast-enhanced MRI can assess angiogenesis, albeit at a resolution of several hundred micrometers [26]. Compared to the tumor core and normal brain tissue, the invasive zone exhibited denser branches and branch points, higher vascular curvature, and increased fractal dimensions. As Hoefnagels et al [27] noted, the invasive zone demonstrates higher vascularization than normal brain tissue, possibly due to GBM-released growth factors that stimulate abnormal, rapid, and tortuous vascular growth. Immature endothelial structures around new blood vessels cause complex branching and defective vascular networks. Moreover, the significantly lower vascular maturity index further confirms the predominance of immature vessels, limiting antitumor drug delivery and causing resistance, with antiangiogenic therapy shown to normalize vessels in both clinical and experimental settings [28, 29]. Quantifying MV normalization could thus evaluate therapeutic effects.

ULM can also assess effective perfusion by detecting red blood cell signals, demonstrating greater orientation variance and perfusion volume in the invasive zone [17, 30], correlating with active cell proliferation and higher oxygen demand, associated with higher PI. However, there was no statistical difference in velocity between the invasive zone and tumor core, possibly due to higher MV density and blood storage in the invasive zone, alongside unbalanced velocity reducing overall mean velocity [31]. Nevertheless, flow velocity remains higher than that in normal brain tissue. No significant variations were observed in vascularity, branch point, curvature, or orientation variance between normal and tumor tissue. This suggests that the core region, being the initial growth site, undergoes inhibited angiogenesis over time, with microvascular density resembling that of normal brain tissue [29]. Branch points primarily reflect the vascular network’s connectivity and are not definitively indicative of neovascularization within tumors. New vessel formation in tumors arises from endothelial cell migration and proliferation, leading to increased branches. Existing vessel extension and regeneration sufficiently meet the tumor’s blood needs without significantly altering branch points [27, 32]. Blood vessels in GBM are typically malformed and irregular, frequently undergoing passive expansion to meet the increased blood flow demands associated with tumor growth rather than improving vascular functionality or efficiency through increased curvature [20, 21]. In normal brain tissue, the vascular system generally retains its structural and directional integrity. During tumor growth, vessels may orient toward the tumor center or well-supplied regions; however, this change does not differ significantly from normal brain tissue [33].

ULM provided a robust visualization method for quantifying vascular parameters. As Seo et al [34] noted, CT can visualize the MV structure of the invasive zone. Our comparative analysis of GBM invasive zone vasculature using micro-CT found that ULM showed significantly higher vascular density, branches, and fractal dimensions, indicating its superior resolution. The tumor core exhibited similar trends. However, no significant differences were observed in branch points and curvature, likely due to ULM’s sensitivity to tiny distal branches, with no difference in the proximal main trunk [34, 35]. Terminal MV often lacks mature supporting structures like endothelial cells, maintaining the texture of the proximal vascular skeleton and producing no significant difference in curvature [36]. SEM comparisons revealed no statistical differences in microstructure but significant differences in curvature, possibly due to vessel displacement during sample dehydration and drying [24, 35]. Overall, this indicates ULM’s practical application potential. Further research is needed to track dynamic changes in the MV system and analyze its role in disease progression in the invasive zone.

Compared with the histopathological reference standard of coregistered brain slices, hematoxylin, and eosin staining confirmed similar ranges. Immunofluorescence highlighted MV outside cell nuclei in merged images, indicating ULM accurately represented untreated MV [33]. Tumor cell proliferation, supported by sufficient local blood supply, creates a vicious cycle as higher PI demands more blood volume [20]. CD31, a marker of vascular endothelium, and Ki67, a marker of cell proliferation, both correlate well with vascular density in ULM. However, the fractal dimension negatively correlated with the VMI-H, possibly because MV in the invasive zone is in an early formation stage, lacking mature myofiber protein around the lumen, leading to disordered arrangements and complex vascular networks [37]. It’s important to note that differences in sampling volume between ULM and histopathological sections may explain some discrepancies, as the ultrasound beam width measures hundreds of micrometers while histopathological sections are 5-μm thick [21].

Although micro-CT and SEM validate ULM’s feasibility, accurately quantifying vascular reconstruction remains challenging without an *in vivo* imaging modality matching the reference standard’s resolution. These methods lack dynamic and longitudinal imaging crucial for monitoring tumor progression and treatment response. Nevertheless, our study offers a complementary MV-scale technique for visualizing vascular responses and evaluating treatment changes. Future research should focus on dynamic MV quantification. As an additional limitation, isoflurane’s dose-dependent vasodilatory effect on cerebral blood flow is a confounding factor, though some reports indicate no correlation between blood flow and ULM indicators [38], reducing the likelihood that cardiovascular parameter differences explain the ULM-related findings in this study. Moreover, craniotomy and prolonged imaging times can induce physiological changes in animals, potentially blurring or exacerbating baseline differences in the vascular system across different regions. Lastly, this approach may not be suitable for studying isocitrate dehydrogenase-mutant gliomas due to less prominent neovascularization [39].

In conclusion, we propose a ULM-based strategy for high-resolution, whole-brain MV-scale reconstruction, offering the potential for non-invasive assessment of GBM. We anticipate that ULM could play a valuable role in the dynamic monitoring and early evaluation of GBM treatment. Our results suggest this method provides a unique approach to investigating potential vascular mechanisms in both structural and functional aspects, offering new insights for tailored GBM management by accurately assessing tumor vascular normalization and developing new applications (Supplementary Fig. S2).

## Supplementary information

**Additional file 1:** **Supplementary Table S1.** MRI scan parameters for each sequence. **Supplementary Table S2.** Number of scan slices for each rat. **Supplementary Table S3.** Comparison of ULM parameters in tumor area, invasive zone, and normal brain area. **Supplementary Table S4.** Comparison of ULM and micro-CT in the invasive zone. **Supplementary Table S5.** Comparison of ULM and micro-CT in the tumor area. **Supplementary Table S6.** Comparison of SEM and ULM. **Supplementary Table S7.** Histopathological comparison between tumor area and invasive zone. **Supplementary Table S8.** Correlation between ULM and histopathology. **Supplementary Fig. S1.** Comparison of ULM and micro-CT in the tumor area: A. Segmentation results from micro-CT and ULM: visualization of vascular skeleton, branches, and branch points in the tumor area; B. Comparison of stuctural parameters between micro-CT and ULM. **Supplementary Fig. S2.** ULM application scheme.

## Data Availability

The datasets used and/or analyzed during the current study are available from the corresponding author upon reasonable request.

## Abbreviations

CH: Capillary hemodynamics
CP: Capillary pattern
GBM: Glioblastoma multiforme
Micro-CT: Micro-computed tomography
MRI: Magnetic resonance imaging
PI: Proliferation index
SEM: Scanning electron microscopy
ULM: Ultrasound localization microscopy
VD-H: Histopathological vascular density
VMI-H: Histopathological vascular maturity index
