# Supplementary material for: Microvascular heterogeneity exploration in core and invasive zones of orthotopic rat glioblastoma via ultrasound localization microscopy
Source: Eur Radiol Exp. 2025 Mar 5;9:30. doi: 10.1186/s41747-025-00555-4 (PMC11882483; doi:10.1186/s41747-025-00555-4)
Supplement: Supplementary file 1 — Additional file 1: Supplementary Table S1. MRI scan parameters for each sequence. Supplementary Table S2. Number of scan slices for each rat. Supplementary Table S3. Comparison of ULM parameters in tumor area, invasive zone, and normal brain area. Supplementary Table S4. Comparison of ULM and micro-CT in the invasive zone. Supplementary Table S5. Comparison of ULM and micro-CT in the tumor area. Supplementary Table S6. Comparison of SEM and ULM. Supplementary Table S7. Histopathological comparison between tumor area and invasive zone. Supplementary Table S8. Correlation between ULM and histopathology. Supplementary Fig. S1. Comparison of ULM and micro-CT in the tumor area: A. Segmentation results from micro-CT and ULM: visualization of vascular skeleton, branches, and branch points in the tumor area; B. Comparison of stuctural parameters between micro-CT and ULM. Supplementary Fig. S2. ULM application scheme. [file 41747_2025_555_MOESM1_ESM.pdf]

Microvascular heterogeneity exploration in core and invasive zones of orthotopic rat glioblastoma via Ultrasound Localization Microscopy

ELECTRONIC SUPPLEMENTARY MATERIAL

Supplementary Table S1 MRI scan parameters for each sequence

Parameters	T2	FLAIR	DWI	CE
Echo time (msec)	92.96	160.96	71	11.38
Repetition time (msec)	3963	8000	3902	1143
Field of view (mm)	60	60	80	35
Slice thickness (mm)	1.0	1.3	1.1	1.0
Slices	22	22	22	22
Slice gap (mm)	0	0	50	0
Laser flip angle (°)	90	90	90	90
Cohesion flip angle (°)	140	150	150	150
Bandwith (Hz)	200	220	960	200

CE Contrast enhanced, *DWI* Diffusion-weighted imaging, *FLAIR* Fluid-attenuated inversion recovery

Supplementary Table S2 Number of scan slices for each rat

Model	Number of slices
Rat1 (micro-CT)	3
Rat2	4
Rat3	5
Rat4	4
Rat5	5
Rat6 (SEM)	3

SEM Scanning electron microscopy

Supplementary Table S3 Comparison of ULM parameters in tumor area, invasive zone, and normal brain area

**Parameters**	**Tumor, N=21 (95%CI)***	***-p1***	**Infiltration, N=21 (95%CI)***	***p2***	**Normal, N=21 (95%CI)***	***p3***
Diameter (μm)‡	34.91 (31.55~38.27)	0.012§	47.41 (41.50~53.32)	<0.001§	25.84 (23.58~28.10)	0.001§
Vascularity (%)†	10.23 (8.54~11.91)	<0.001§	21.27 (19.11~23.42)	<0.001§	12.30 (10.32~14.29)	0.367
Branch (/mm²)‡	35.12 (28.49~41.55)	<0.001§	135.04 (112.75~157.33)	<0.001§	59.55 (46.03~73.07)	0.040§
Branch point (/mm²)‡	11.20 (8.82~13.58)	<0.001§	51.40 (42.03~60.77)	<0.001§	19.61 (13.71~25.51)	0.104
Curvature‡	1.1356 (1.1244~1.1477)	<0.001§	1.1953 (1.1811~1.2116)	<0.001§	1.1314 (1.1210~1.1428)	0.631
Fractal dimension‡	1.3710 (1.334~1.408)	<0.001§	1.5135 (1.4951~1.5319)	<0.001§	1.2772 (1.2284~1.3260)	0.036§
Velocity (mm/sec)‡	35.03 (34.15~35.91)	0.873	34.82 (33.78~35.86)	<0.001§	29.02 (28.29~29.75)	<0.001§
Orientation variance‡	17.00 (14.17~19.83)	<0.001§	26.96 (22.66~31.26)	0.001§	18.57 (16.51~20.63)	0.528
Blood flow (μl)‡	1227.83 (1093.18~1362.48)	0.033§	1658.42 (1429.88~1886.96)	<0.001§	750.48 (679.59~821.37)	<0.001§

*Data are given with 95% confidence intervals in parentheses.

†*P* values for vascularity were obtained by ANOVA.

‡*P* values for other parameters were obtained by Kruskal Wallis-H-test.

§Statistical significance.

p1: tumor vs infiltration; *p2*: infiltration vs normal; *p3*: normal vs tumor

CI Confidence interval, *N* Number of clices, *ULM* Ultrasound localization microscopy

Supplementary Table S4 Comparison of ULM and micro-CT in the invasive zone

**Parameters**	**ULM, N=3 (95%CI)***	**Micro-CT, N=3 (95%CI)***	***p***
Diameter (μm)†	31.90 (20.84~42.96)	49.93 (26.19~73.67)	0.041§
Vascularity (%)†	19.60 (8.06~31.14)	10.65 (8.35~12.95)	0.031§
Branch (/mm²)†	105.79 (27.96~183.62)	42.96 (4.04~81.88)	0.036§
Branch point (/mm²)†	39.04 (1.23~76.85)	15.45 (2.83~28.07)	0.064
Curvature†	1.1655 (1.1546~1.1840)	1.1743 (1.1543~1.2033)	0.643
Fractal dimension†	1.5245 (1.4429~1.6016)	1.2959 (1.1775~0.4143)	0.002§

*Data are given with 95% confidence intervals in parentheses.

†*P* values for comparison between ULM and micro-CT by t test.

§Statistical significance.

CI Confidence interval, *N* Number of clices, *ULM* Ultrasound localization microscopy

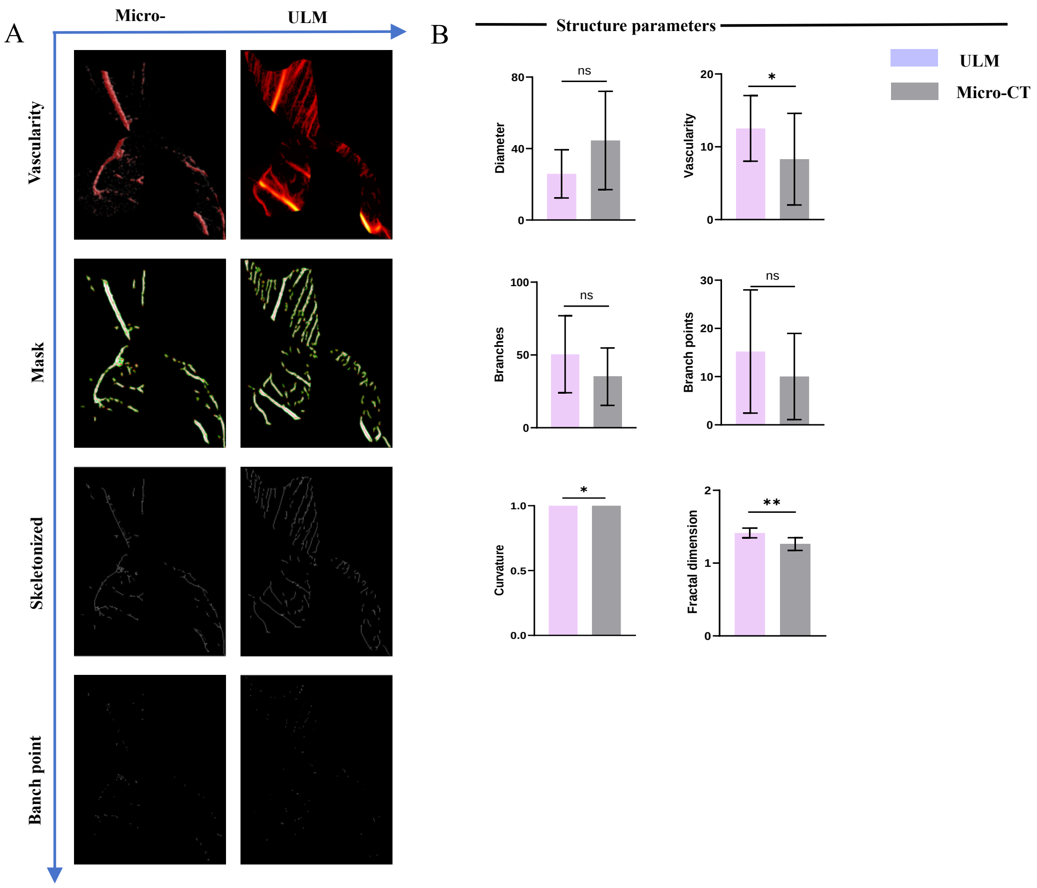

Supplementary Fig. S1 Comparison of ULM and micro-CT in the tumor area: A. Segmentation results from micro-CT and ULM: visualization of vascular skeleton, branches, and branch points in the tumor area; B. Comparison of stuctural parameters between micro-CT and ULM.

Supplementary Table S5 Comparison of ULM and micro-CT in the tumor area

Parameters	ULM, N=3 (95%CI)*	Micro-CT, N=3 (95%CI)*	*p*
Diameter (μm)†	25.88 (12.40~39.36)	44.54 (17.05~72.03)	0.059
Vascularity (%)†	12.53 (8.02~17.04)	8.30 (2.01~14.59)	0.078
Branch(/mm²)†	50.50 (24.02~76.98)	35.10 (15.39~54.81)	0.115
Branch point (/mm²)†	14.38 (-1.62~30.38)	10.03 (1.09~18.97)	0.227
Curvature†	1.1216 (1.1083~1.1334)	1.1726 (1.1487~1.1959)	0.030§
Fractal dimension†	1.4142 (1.3481~1.4803)	1.2620 (1.1749~1.3491)	0.004§

*Data are given with 95% confidence intervals in parentheses.

†*P* values for comparison between ULM and micro-CT by t test.

§Statistical significance.

CI Confidence interval, *N* Number of clices, *ULM* Ultrasound localization microscopy

Supplementary Table S6 Comparison of SEM and ULM

**Parameters**	**SEM, N=3 (95%CI)***	**ULM, N=3 (95%CI)***	***p***
Diameter (μm)†	39.67 (16.01~63.33)	52.63 (16.18~29.09)	0.379
Vascularity (%)†	2.55 (-0.22~5.33)	5.10 (2.46~7.74)	0.079
Branch (/mm²)†	18.75 (-6.75~44.25)	9.20 (-9.88~28.28)	0.377
Branch point (/mm²)†	3.44 (1.80~5.09)	3.80 (-0.50~8.10)	0.813
Curvature†	1.1296 (1.0512~1.2083)	1.3799 (1.2496~1.4862)	0.019§
Fractal dimension†	1.2328 (1.1768~1.2888)	1.1965 (1.1613~1.2317)	0.131

*Data are given with 95% confidence intervals in parentheses.

†*P* values for comparison between SEM and ULM by t test.

§Statistical significance.

N Number of clices, *SEM* Scanning electron microscopy, *ULM* Ultrasound localization microscopy

Supplementary Table S7 Histopathological comparison between tumor area and invasive zone

**Histopathology**	**Tumor, N=21 (95%CI)***	**Infiltration, N=21 (95%CI)***	***p***
VD-H (%)†	2.66 (1.65~3.67)	5.34 (3.45~7.23)	0.006 §
PI (%)†	4.49 (1.56~7.42)	6.66 (3.65~9.67)	0.030 §
VMI-H (%)†	20.04 (11.55~28.53)	7.60 (5.26~9.944)	0.019 §

*Data are given with 95% confidence intervals in parentheses.

†*P* values for comparison between tumor and infiltration by t test.

§Statistical significance.

CI Confidence interval, *N* Number of clices, *PI* Proliferation index, *VD-H* Histopathological vascular density, *VMI-H* Histopathological vascular maturity index

Supplementary Table S8 Correlation between ULM and histopathology

Quantification Parameter	VD-H, N=21 (95%CI)*	PI, N=21 (95%CI)*	VMI-H, N=21 (95%CI)*
Structural			
Diameter (μm)	-0.014 (-0.473,0.458)	0.960 (0.860,0.988)§	0.312 (-0.258,0.682)†
Vascularity (%)	0.781 (0.336,1.000)§	-0.077 (-0.537,0.407)	-0.444 (-0.684, -0.088)†§
Branch (/mm²)	0.300 (-0.177,0.661)	-0.136 (-0.560,0.304)	0.193 (-0.278,0.627)†
Branch point (/mm²)	0.432 (-0.005,0.733)	-0.110 (-0.500,0.310)	-0.019 (-0.457,0.429)
Curvature	-0.106 (-0.545,0.383)	0.438 (0.024, 0.685)§	0.227 (-0.197,0.632)
Fractal dimension	0.161 (-0.299,0.561)	-0.152 (-0.650,0.361)	-0.933 (-0.985, -0.911)†§
Hemodynamics			
Velocity (mm/sec)	0.162 (-0.260,0.607)	0.487 (0.171,0.715)§	-0.015 (-0.498,0.439)†
Orientiation variance	0.247 (-0.220,0.680)	-0.194 (-0.558,0.251)	-0.155 (-0.526,0.271)
Functional			
Blood flow (μl)	-0.043 (-0.529,0.436)	0.858 (0.823,0.978)§	0.286 (-0.293,0.681)†

*Data are given with 95% confidence intervals in parentheses.

†The correlation r-values were calculated using Pearson's method, while the remaining values were obtained by Spearman's method.

§Statistical significance.

CI Confidence interval, *N* Number of clices, *PI* Proliferation index, *VD-H* Histopathological vascular density, *VMI-H* Histopathological vascular maturity index

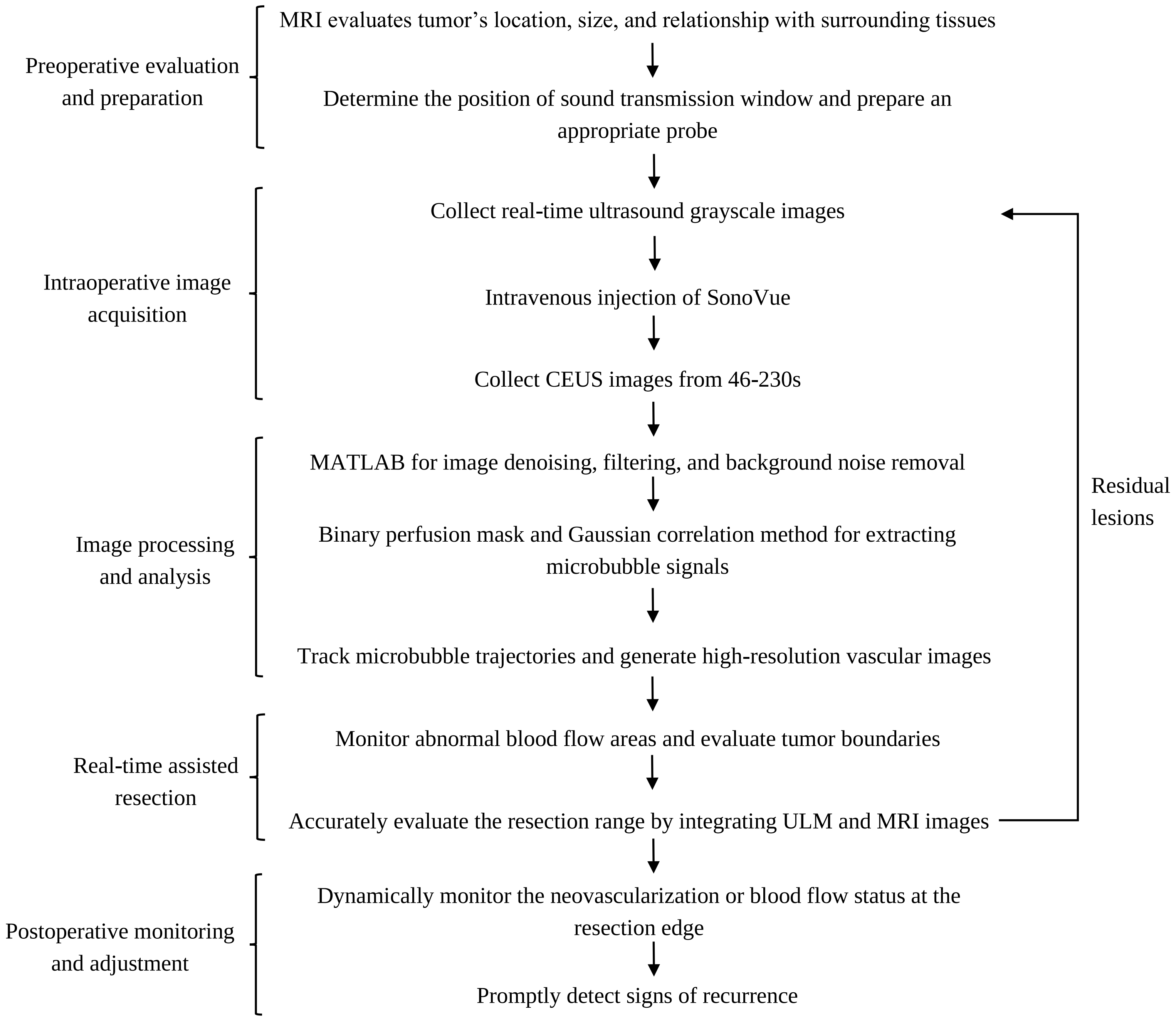

Supplementary Fig. S2 ULM application scheme
